# In Situ Single-Crystal X‑ray Diffraction Studies of an Anomalous Nitric Oxide Adsorption in a Partially Activated Metal–Organic Framework

**DOI:** 10.1021/jacs.5c10395

**Published:** 2025-08-14

**Authors:** Russell M. Main, Romy Ettlinger, Tia K. Tajnšek, Deborah A. Brako-Amoafo, Maximillian G. Stanzione, Morven J. Duncan, Philip Ettlinger, Gaynor B. Lawrence, Mark R. Warren, Christopher J. Heard, Russell E. Morris

**Affiliations:** † EaStCHEM School of Chemistry, Purdie Building, North Haugh, St Andrews KY16 9ST, U.K.; ‡ TUM School of Natural Sciences, Technical University of Munich, Lichtenbergstr. 4, 85748 Garching bei München, Germany; § National Institute of Chemistry, Hajdrihova 19, 1000 Ljubljana, Slovenia; ∥ Department of Physical and Macromolecular Chemistry, Charles Univsersity, Hlavova 8, 12800 Prague 2, Czech Republic; ⊥ Diamond Light Source Ltd, Diamond House, Harwell Science & Innovation Campus, Didcot OX11 0DE, U.K.

## Abstract

Metal–organic frameworks (MOFs), with their high porosities and surface areas, show great utility in the field of gas adsorption. To unlock this porosity, MOFs are generally fully activated by removing all adsorbed guests using high temperatures and low pressures. However, this is energy intensive and can be unfeasible if the MOF is part of a composite, where the maximum temperature of the composite is below the activation temperature. To investigate the effect of activation temperature on adsorption, a series of in situ single-crystal X-ray diffraction (scXRD) studies were performed on Ni-MOF-74 loaded with the gas nitric oxide (NO) under different conditions. These experiments uncovered anomalous adsorption results where partially activated samples adsorb ∼14% more NO per framework material than did the fully activated sample. The scXRD experiments revealed a new NO binding site that is only present if the open metal sites are partially occupied by water molecules. To shed more light on the respective binding of the two different NO sites in Ni-MOF-74, these were studied in situ under different treatment conditions, such as the exposure to vacuum at different temperatures. This study yields insights into the nature of binding sites in MOFs, how these are affected by activation, and helps to pave the way for the improved design of processing conditions.

## Graphical abstract

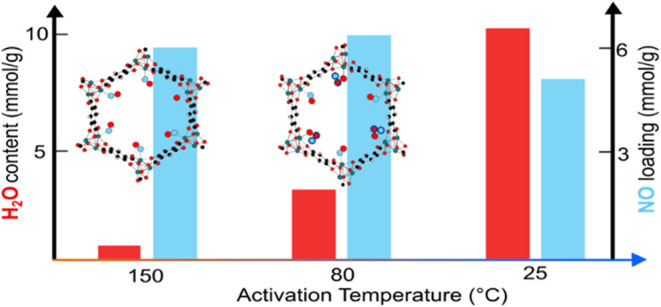

## Introduction

Metal–organic frameworks (MOFs) are a widely studied class of porous materials.^1,2^ They comprise metal ions and clusters and organic linkers connected into porous crystalline frameworks. Their exceptional porosities and tunable chemistries promise many potential applications from the adsorption of gases to zygote gene therapy.^3–7^ One such application is the capture, storage, and release of gases such as nitric oxide (NO). As a member of the toxic NOx family of atmospheric pollutants it is highly desirable to capture NO from the atmosphere.^8^ However, NO is also a biologically active molecule, acting as a neurotransmitter known to have vasodilatory, antithrombotic, wound healing, and antimicrobial properties, and so has potential beneficial uses.^9–11^

In order to unlock their applicability, MOFs are typically activated to gain access to their internal porosity. This generally involves exposing the framework to heat and vacuum conditions to remove all occluded solvent or other guest molecules. In gas adsorption applications, this process is particularly important as maximum adsorption is believed to be possible only when the whole framework porosity is available.^2,12^ However, fully activating samples is energy intensive and keeping them activated may sometimes be difficult, especially in MOFs with open metal sites, which can rapidly readsorb water from the atmosphere.^13^ Strenuous activation conditions can also reduce the cyclability of a material, as the stress imparted from high temperatures and low pressures combined with solvent removal can be detrimental to the framework.^14^ In some flexible MOFs full activation can reduce gas adsorption as it causes the pore to contract reducing accessibility.^15^

In many applications, MOFs cannot be used in their pure powdered form but they can be incorporated with polymers to prepare more functional composite materials.^16–18^ However, the polymer puts its own requirements on the possible activation conditions, with many polymers degrading at the high temperatures needed to fully activate MOFs.^19,20^ More work is needed to examine how milder activation conditions affect the amount and type of gas binding. If gas loading can be maintained in a partially activated sample, it would save energy, improve processability, and increase recyclability.

In situ single-crystal X-ray diffraction (scXRD) is an established technique to study gas binding within MOFs.^21–25^ This technique is typically used to give crystal structures of materials where gas molecules are introduced at high pressure to a sample fully activated under vacuum at high temperatures.^26^ Under these conditions, there is no competition from molecules such as water. In many applications, however, where achieving perfect activation is not possible, the presence of residual water in the MOF is considered detrimental to gas binding.^27,28^

The MOF-74/CPO-27 family of MOFs has a high density of open metal sites, making them ideal for the binding of polar gases such as NO. MOF-74 is formed from 2,5-dihydroxyterephthalic acid and M­(II) ions (Ni, Mg, Zn, Cu, etc.) and crystallizes in the *R*3̅ space group.^23^ It consists of hexagonal channels running parallel to the crystallographic *c*-axis, with open metal sites at each corner of the hexagon in the fully activated material. Fully activated Ni-MOF-74 shows exceptional storage of NO with one NO binding at each open metal site, as has been shown by previous X-ray diffraction experiments. This NO can be released upon exposure to moisture, as water binds preferentially to the open metal site.^29–31^

With the right loading and release procedure, MOF-74 can be utilized to store and deliver NO in suitable quantities and at appropriate rates for medical applications. In previous work to load MOFs with NO, activation at reduced pressures and high temperatures, typically 150 °C or above, was required to remove the metal-bound water and produce the maximum number of open metal sites. The material was then exposed to a NO atmosphere.^32^ To safely remove the material from this atmosphere, the chamber must be subjected to a purge procedure of vacuum and argon cycling. Once removed safely, the sample can be exposed to moisture, for instance, a humid gas stream or liquid water, which releases the NO.^30^ By storing the NO-loaded MOF under dry conditions, the NO can be transported and selectively released at a required location, for instance for antimicrobial applications.^10^ Other fields, such as the capture and degradation of NOx also rely on fully activating a MOF before it can be used. Fully activated MOF-74 can capture NOx from the atmosphere.^33–35^ However, despite this extensive work on adsorption, it is less well studied how partially activated samples, which are less energy intensive to produce, can adsorb these pollutants.

In this work, we examine how the adsorption of N_2_ and NO into Ni-MOF-74 is affected by different activation conditions with NO showing unexpectedly high adsorption capacities after partial activation. We used synchrotron X-rays at the Diamond Light Source, U.K., to perform in situ scXRD experiments to understand how NO and water behave in Ni-MOF-74. We study partially activated Ni-MOF-74 that still contains chemisorbed water (i.e., water bound to the metal ions in the framework) to see how this effects NO loading and compare this with the loading of a fully activated sample. The key finding from the work is that the partially activated MOF has an additional binding site where the NO is not connected to the metal but is instead interacting with residual chemisorbed water molecules. This is not possible in a fully activated MOF as there is no such chemisorbed water present. This leads to a surprisingly large amount of NO adsorbed in the partially activated material compared to its fully activated counterpart. The work demonstrates that partial activation, with its lower energy requirement and enhanced compatibility with other thermally sensitive materials, may be beneficial when designing the processing of MOFs.

## Results

### Impact of Different Activation Conditions on the Gas Binding Performance of Ni-MOF-74

The temperature used to activate Ni-MOF-74 is expected to have a strong effect on the guest solvent content and its gas adsorption properties. To examine this, the effect of different activation temperatures was first analyzed with N_2_ adsorption isotherms at 77 K (Figure S-1). These clearly show a reduction in N_2_ adsorption capacity when the activation temperature is decreased from 177 to 25 °C (Figure 1). After activation at 177 and 150 °C, Brunauer–Emmett–Teller (BET) surface areas of 1370 and 1360 m^2^/g, respectively, were determined, which is in line with literature values.^36^ Reducing the activation temperature to 80 °C caused a 24% decrease in the available surface area to 1030 m^2^/g. This indicates that the framework is not fully activated at 80 °C and still contains water in the pores, which in turn reduces the surface area accessible to nitrogen molecules. Reducing the activation further to 50 and 25 °C caused further reductions in the calculated BET surface area to 690 and 470 m^2^/g, respectively. These results indicate, as one would expect, that lowering the activation temperature does not remove all the guest solvent molecules from the pores of the MOF. We describe these materials to be partially activated.

**Figure 1:**
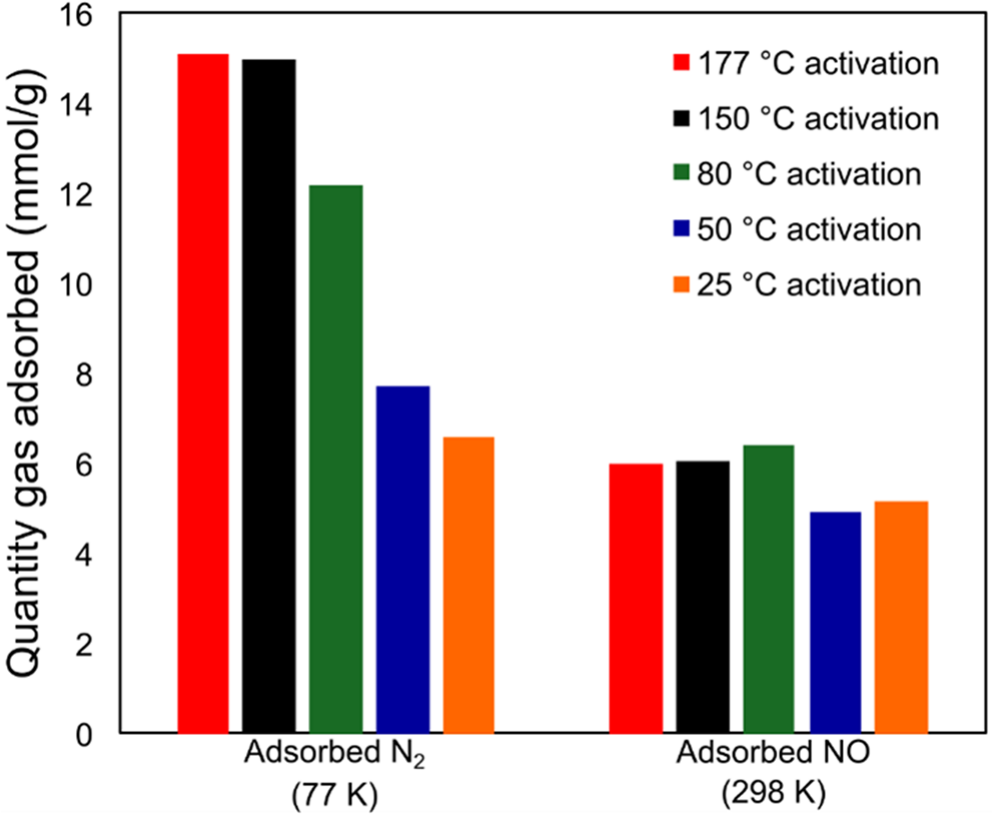
Showing from left to right the total quantities of N_2_ adsorbed at 77K and of NO adsorbed at 1 bar NO at 25 °C after activation at different temperatures: 177 °C (red), 150 °C (black), 80 °C (green), 50 °C (blue), and 25 °C (orange). Note that the amount of water in the samples will be different for each activation condition (Table 1).

Sorption isotherms with NO show a different and surprising trend. Using a gravimetric gas adsorption rig, the ability of Ni-MOF-74 to bind NO after overnight activation under vacuum (10^–4^ mbar) at different temperatures (177, 150, 80, 50, and 25 °C) was examined. The amount of NO adsorbed at 1 bar was 6.2 ± 0.2 mmol/g for the samples activated at 177, 150, and 80 °C. This is in good agreement with the theoretical amount of NO loading, 6.4 mmol/g, that assumes one NO molecule binds to each metal site in a fully activated material. Interestingly, reducing the activation temperature had no significant effect on the absolute amount of NO adsorbed. Reducing the activation temperature to 50 and 25 °C caused a reduction in NO adsorption to 5.0 ± 0.2 mmol/g.

This result is surprising as for partially activated MOFs, we expect that there is still water occupying the metal site at the lower activation temperatures, theoretically restricting access for NO binding.^32^ For example, the mass loss on activation at 177 °C is 40.2 wt %, which corresponds to loss of 5.8 molecules of water per Ni—this agrees closely with other studies of the full activation of Ni-MOF-74.^37^ Activation at 80 °C results in a mass loss of 36.8%, corresponding to a residual 0.5 molecules of water per Ni atom remaining in the material (i.e., loss of 5.3 molecules per Ni). This agrees very well with the crystal structure of the MOF after such an activation, as reported below. Activation under vacuum at 25 °C results in a 28.6% mass loss, corresponding to a residual 1.8 molecules of water per Ni atom remaining in the material; this is consistent with all the nickel atoms coordinating to a water molecule and some of the stronger uncoordinated water adsorption sites being occupied. Again, this is in agreement with the previous work.^37^ From this information, we can then calculate the number of moles of framework (including residual water) present in a gram of material in each of the experiments shown in Figure 1. Taking the basic formula of the MOF as Ni_2_(C_8_O_6_H_2_)·*x*H_2_O where *x* = 0 for a fully activated sample, *x* = 1 for a sample activated at 80 °C and *x* = 3.6 for a sample activated at 25 °C we calculate the number of moles of NO adsorbed per mole of framework material as 1.87, 2.13, and 1.90 for activation at 177, 80, and 25 °C respectively. This suggests that partial activation at 80 °C is the best of these conditions, with ∼14% more NO being adsorbed per formula unit of the MOF material than for a fully activated sample (Table 1). This anomalous adsorption behavior is the focus of the following structural study. Even though NO is known to interact very differently to N_2_,^38,39^ it is surprising that the NO adsorption capacity is not negatively affected by this water content. This suggests that the open metal site might not be the only possible binding site for NO and that more NO is adsorbed than would be expected based on the availability of open metal sites.

**Table 1:** Outlining how the Different Activation Conditions Affect Water Content, Surface Area, and Total NO Loadings of Ni-MOF-74

activation temperature (°C)	formula after activation	BET surface area (m^2^/g)	NO adsorbed per gram of material (mmol)	NO adsorbed per mol of framework (mmol)
177	Ni_2_(C_8_O_6_H_2_)	1370	6.0	1.87
150	Ni_2_(C_8_O_6_H_2_)·0.2(H_2_O)	1360	6.1	1.90
80	Ni_2_(C_8_O_6_H_2_)·1(H_2_O)	1030	6.4	2.13
50	Ni_2_(C_8_O_6_H_2_)·1.7(H_2_O)	690	4.9	1.70
25	Ni_2_(C_8_O_6_H_2_)·3.3(H_2_O)	470	5.1	1.90

### In Situ scXRD Investigation

To investigate the NO binding mechanism in more detail, a series of in situ scXRD studies were performed using the synchrotron source at Diamond Light Source. Mimicking the established laboratory activation conditions, Ni-MOF-74 was subject to either high temperature conditions at 177 °C or mild conditions at 80 °C and the amount of metal-bound water (O_w_) was determined from the diffraction data. The samples were then loaded with NO and the position of NO binding was determined.

After heating to 177 °C, the occupancy of O_w_ was reduced to less than 10%. This is similar to previous results where it has been shown to be difficult to reach zero water occupancy from these relatively large crystals in the gas cell on the X-ray diffractometer.^32^ On cooling down to room temperature, the occupancy of O_w_ increases to 17(1)%. This is because of readsorption of water trapped within the experimental equipment, as has been noted previously.^23,32^ This is equivalent to approximately one-sixth of the open metal sites still having coordinated water, as shown in Figure 2b.

**Figure 2:**
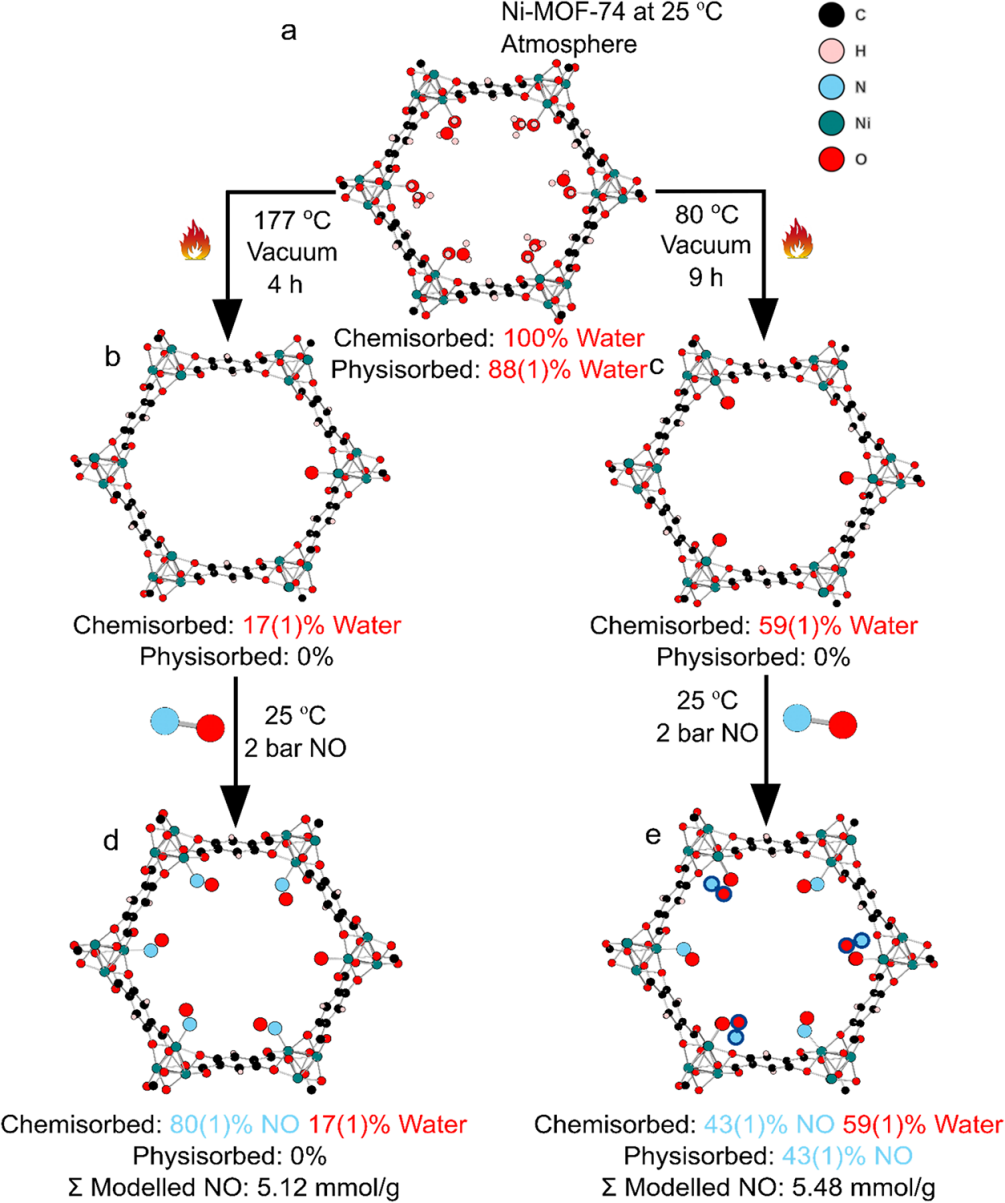
Ball and stick figures of Ni-MOF-74 down the crystallographic *c*-axis schematically show occupancies under different conditions. (a) Room temperature and atmospheric pressure—note that the center of the pores will also contain further water molecules but these are not shown for clarity. (b) Activated at 177 °C under vacuum for 4 h and cooled back to 25 °C. (c) Activated at 80 °C under vacuum for 9 h and cooled back to 25 °C. (d) Part (b) exposed to 2 bar NO at 25 °C. (e) Part (c) exposed to 2 bar NO at 25 °C. The physisorbed NO molecules are highlighted with a blue outline. Note that for species with occupancy less than 100% (e.g., the coordinated water in (b)), the figure only shows one possible position of that species. The crystal structure will be an average of all possible positions.

To replicate the mild activation conditions, a single crystal of Ni-MOF-74 was activated at 80 °C for 9 h under dynamic vacuum (10^–6^ mbar), this resulted in a metal bound water occupancy (O_w_) of 53(1)%. Cooling the sample to 25 °C increased this slightly to 59(1)% (Figure 2c, Table S-1). Under this protocol, it appears that these conditions are, as expected, insufficient to fully activate the MOF and that only half the metals are uncoordinated. This result is in line with the N_2_ adsorption experiments discussed previously.

Exposing the crystal shown in Figure 2a (O_w_ occupancy = 17(1)%) to a 2 bar NO atmosphere causes NO to bind at the metal site with 80(1)% occupancy (Figure 2d).^32^ The total amount of NO modeled amounts to 5.12 mmol/g of MOF, which is less than that observed in the adsorption experiments suggesting that there may be some additional free NO in the pore that cannot be modeled with scXRD.

For the crystal shown in Figure 2c (O_w_ occupancy = 59(1)%), NO was introduced to the system at different pressures from 0.01 bar up to 2 bar (Figures 2e, 3 and Table S-2, Figure S-2). It was possible to model the loaded system with NO molecules bound to the remaining open-metal sites. However, difference Fourier maps revealed extra electron density within the pores that could be modeled as an additional physisorbed NO molecule located close to the wall of the pores between adjacent metal sites (here we use physisorbed to describe a molecule not chemically bonded to a metal center and chemisorbed to describe one that is bonded). Both coordinated (chemisorbed) and physisorbed NO sites increased in occupancy with increasing pressure following a type I isotherm shape, reaching maximum occupancies at about 0.4 bar (Figure 3). NO is observed to bind to the physisorbed site only when there are significant amounts of water present at the metal site. Compared to the activation at 177 °C (O_w_ < 10%), this physisorbed site was not observed. The total amount of modeled chemi- and physisorbed NO of 5.48 mmol/g is approximately equal to the total amount of chemisorbed NO that was modeled for the sample activated at 177 °C. The identification of this additional physisorbed site for NO in partially activated Ni-MOF-74 is surprising and indicates that the reduced thermal processing of the MOF is not as disadvantageous as one might have expected considering the information previously known about NO adsorption in this MOF.

**Figure 3:**
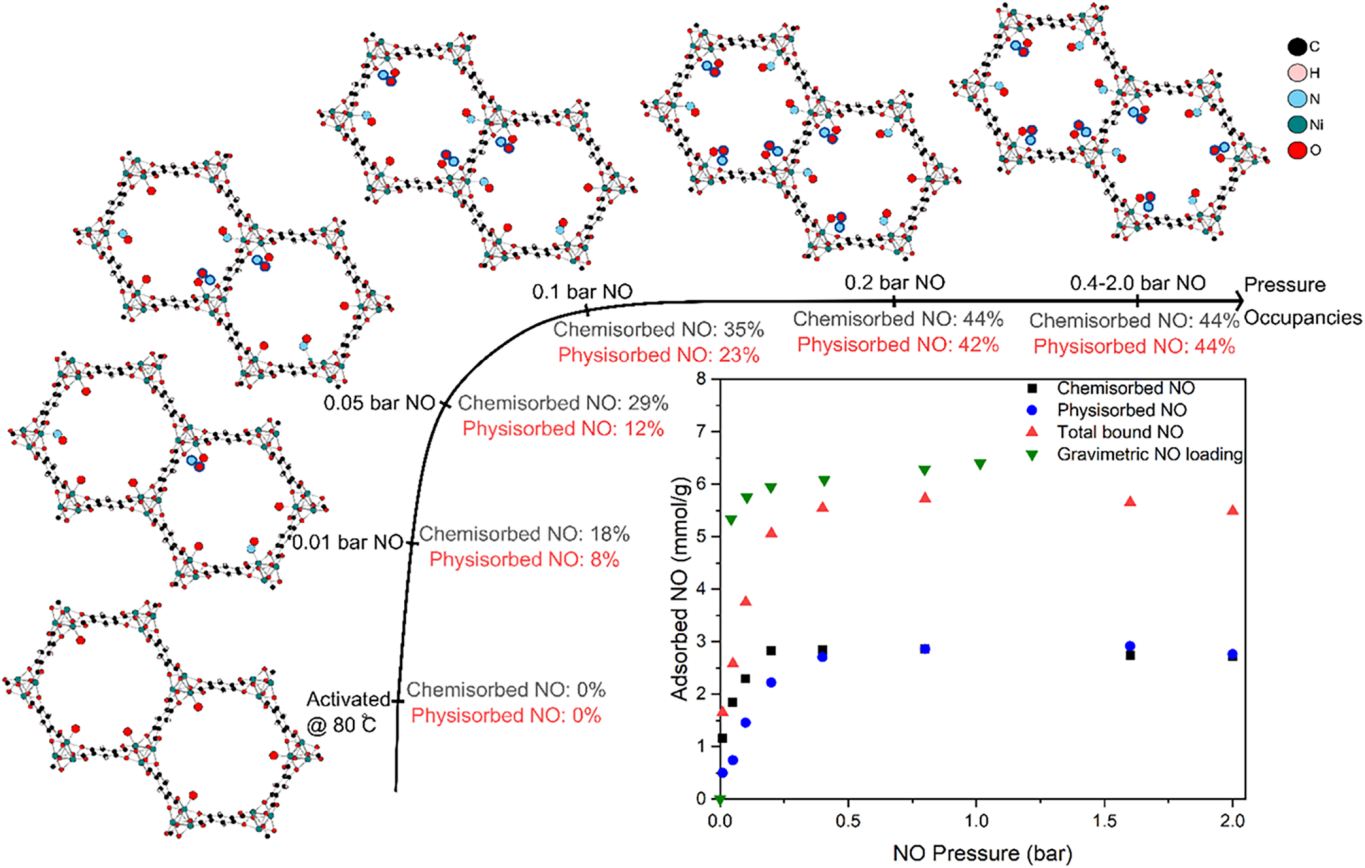
NO loading isotherms at 25 °C of Ni-MOF-74, after mild activation at 80 °C, obtained with the scXRD data: Modeled chemisorbed NO (black squares), modeled physisorbed NO (blue circles), and total modeled NO (red triangles), as well as gravimetric loading data (green triangles). Ball and stick schematic representations of the gas occupancies within Ni-MOF-74 for each pressure point obtained. The error in each NO occupancy is around 1%.

The total amount of NO modeled in the two sites is similar to that obtained by gravimetric analysis of NO loading. In addition, the adsorption isotherm obtained from the gravimetric studies matches reasonably well in terms of the total amount adsorbed and the shape of the isotherm derived from the scXRD refinements (Figure 3). The slight discrepancy between the scXRD model and the gravimetric data can be attributed to unmodeled NO in the middle of the pore, explored below. The fact that we can develop robust adsorption isotherms from scXRD confirms previous work of others on different MOF systems where mostly powder X-ray diffraction has been used to provide information that helps to understand the molecular processes ongoing during an adsorption experiment, going beyond the usual identification of different sites in the material to provide site-specific variation of occupancy at different pressures.^40–43^

A shorter and less thorough activation procedure at 77 °C for 3 h under dynamic vacuum (10^–6^ mbar) produced a similar residual O_w_ occupancy, 54(1)% (Figure 4, Table S-4). NO loaded into this sample to 0.08 bar at 77 °C resulted in NO binding to the open metal sites with 25(1)% occupancy; therefore, NO has bound into nearly half of the remaining open metal sites. On cooling the sample to 25 °C there are many changes to the binding. First, some water recondenses at the open metal sites along with the NO, leading to 72(3)% O_w_ and 36(3)% NO. The additional uptake of water in a NO atmosphere is not entirely unexpected under these conditions due to the water remaining in the experimental setup. Similar behavior has been seen before with CO and NO.^23^ The physisorbed site was observed with an occupancy of 58(1)%. On increasing the pressure incrementally to 2 bar, the occupancies of NO for the chemisorbed site are relatively unchanged, and the physisorbed site follows the shape of a type I adsorption isotherm (Figure S-2). At 2 bar, the total occupancy of NO in the physisorbed site is 69(1)%. The higher amount of physisorbed NO matches the higher amount of chemisorbed water, indicating that the NO is binding to this water.

**Figure 4:**
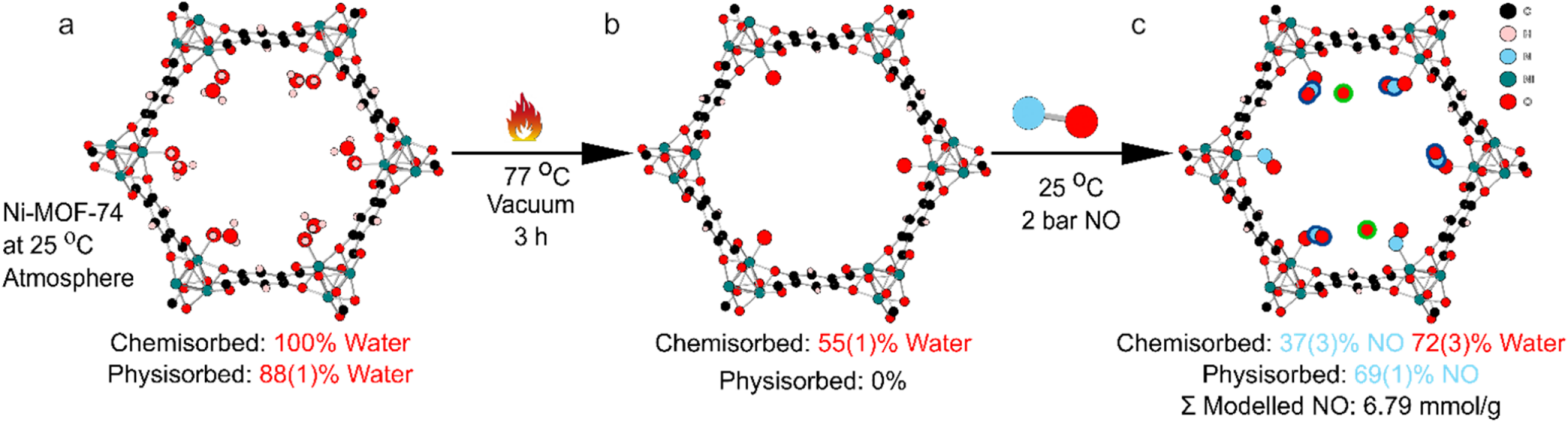
Ball and stick figures of Ni-MOF-74 down the crystallographic *c*-axis under different conditions. (a) Room temperature and pressure. (b) Activated at 77 °C under vacuum for 3 h. (c) Part (b) exposed to 2 bar of NO at 25 °C. Physisorbed NO is highlighted in blue. Further electron density modeled as an oxygen atom is highlighted in green.

Despite the effects of scattering from the gas cell, the lack of twinning in this data set allows for high-quality refinement. Not only does this allow for more of the disorder in the metal-bound NO to be modeled (Figures S-4 and S-5), it also allows tentative modeling of physisorbed NO deeper in the pore—we call this the second physisorbed site. A region of high electron density can be modeled with an isotropic oxygen atom of up to 42(1)% occupancy (at 1 bar NO) and follows a type I isotherm on loading at different pressures (Figure S-2). It is too disordered to be accurately modeled as a molecular species, but it may explain why the model after mild activation (59% O_w_) does not fully match the gravimetric NO loading: there is some additional NO bound weakly in the center of the pore. Furthermore, this site is only observable in scXRD when there is water or physisorbed NO present, which suggests that extra interactions between adsorbed species are important. This would explain why this site is not seen in fully activated samples (<10% O_w_). A similar site has been observed before with SO_2_ adsorption experiments in the presence of water.^24^

The unmodelled gas found within the center of the pore can be approximated by calculating the excess electron density with the Olex mask command using a 1.2 Å probe.^44^ The calculated electron density for the crystal activated at 80 °C for 9 h shows a type I isotherm on loading (Figure S-3). However, the sample activated at 77 °C for 3 h did not show the same effect and is much more variable. In addition, the NO-loaded crystal after activation at 177 °C shows 0 excess electronic density before and after loading. Therefore, although informative, no quantitative information can be obtained from this technique due to the diffuse diffraction from the gas cell and crystal degradation affecting the value.

### Exploring the New Binding Site

The potential binding interactions between the physisorbed NO and the metal-bound water are depicted in Figure 5. The nitrogen atom is somewhat disordered, and so this is just an average position, and so the specific interatomic distances are not necessarily representative of any real interaction distances between two atoms; they are still informative to some degree. The interactions appear to be (1) between the oxygen and the water hydrogen with a bond distance of 2.41(1) Å, well within range for a hydrogen bond^45^ and (2) from the nitrogen to a framework oxygen with a shorter average interatomic distance of 1.7(1) Å. This nitrogen site, however, is likely moving throughout the pore environment and had to be constrained in the low pressure experiments to obtain a stable refinement. It is possible to imagine a simple dipole chain existing between these four atoms. The H is δ+, the O is δ−, the N is δ+, and the framework O is δ−. This van der Waals type of interaction may explain why water is needed at the binding site. If NO is present instead of water, the O does not have the correct dipole moment to form this interaction.

**Figure 5:**
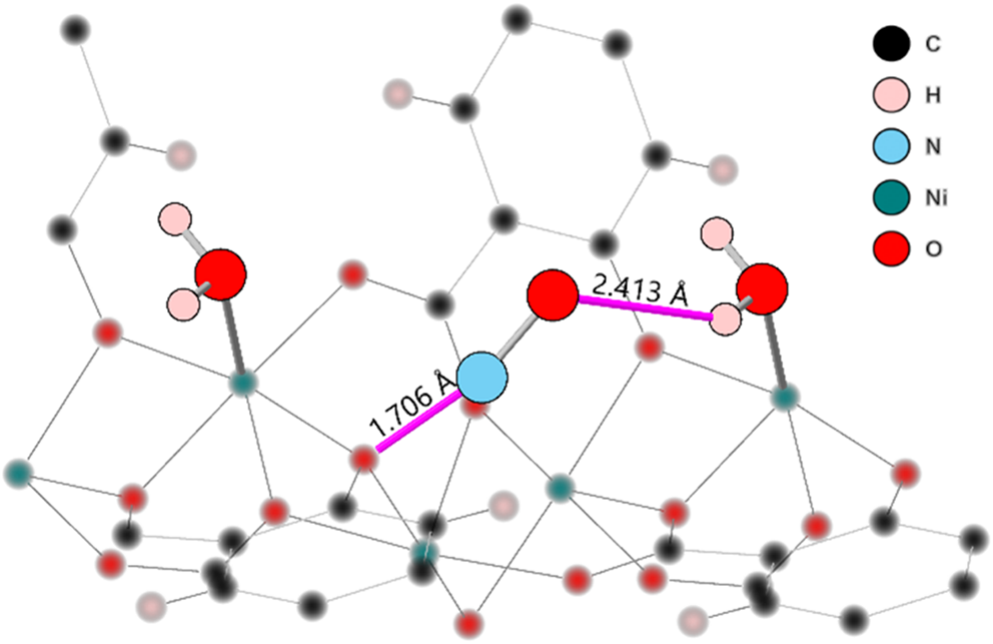
Ball and stick model of NO-loaded Ni-MOF-74 showing the interaction distances of the physisorbed NO with metal-bound water and the framework. The framework is shown to be blurred to highlight the adsorbed molecules.

### In Situ scXRD Studies of NO Desorption

For gas delivery applications, for example, in healthcare, the bound NO ideally should remain in place after exposure to vacuum (stored in the MOF) and only be released on exposure to water through a cooperative exchange mechanism. To shed more light on the stability of the NO-loaded samples, they were subjected to different vacuum and temperature conditions. In the gas cell line, we were able to mimic the purge process undertaken in the laboratory, where excess NO is removed under vacuum/argon cycling. Specifically, we subjected the NO-loaded crystal (sample after mild activation at 80 °C and with 59% O_w_) to three rounds of vacuum/argon cycling, which is sufficient to remove excess gaseous NO from the system. This process did not change the occupancy of chemisorbed or physisorbed NO. Maintaining the sample under dynamic vacuum (10^–2^ mbar) for a further 4 h and increasing the temperature to 50 °C resulted in a reduction in the occupancy of both sites: to 82(1)% for the chemisorbed site and to 14(1)% for the physisorbed site (Figure 6a). It is not possible from this data to determine the individual amounts of NO or water at the metal site during this procedure.

**Figure 6:**
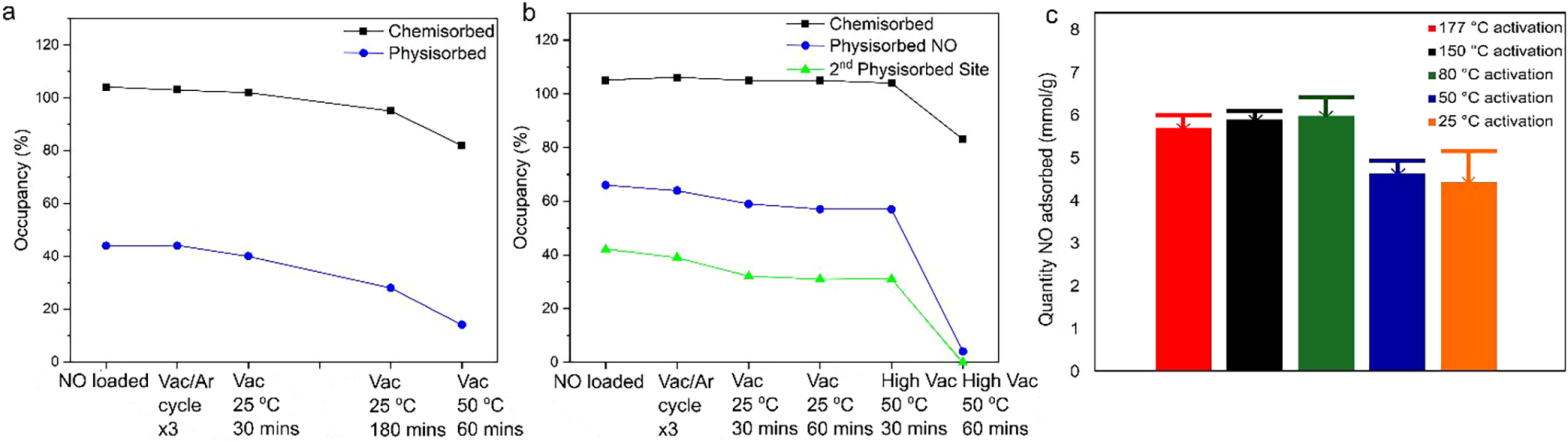
(a) Plot showing how the occupancy of chemisorbed (black) and physisorbed (blue) sites in NO-loaded Ni-MOF-74 (53% O_w_) varies with different vacuum and temperature conditions. (b) Plot showing how, in NO-loaded Ni-MOF-74 (63% O_w_), the total chemisorbed occupancy (black), physisorbed NO occupancy (blue), and second physisorbed site (green) are affected by different vacuum and temperature conditions. (c) Gravimetric NO adsorbed after loading at 1 bar of NO (marked with “box drawings light down and horizontal”) followed by vacuum treatment for 30 min at 10^–1^ mbar after activation at different temperatures: 177 °C (red), 150 °C (black), 80 °C (green), 50 °C (blue), and 25 °C (orange).

Calculating the excess electron density using the Olex mask command (similar to the Squeeze routine in other crystallographic software) with a 1.2 Å probe^44^ throughout the vacuum process shows a constant reduction in electron density (Figure S-3). However, this value never reaches 0 again, likely due to crystal degradation during the activation, loading, and vacuum conditions.

Performing three rounds of vacuum/argon cycling on the NO-loaded crystal with 72(3)% O_w_ (sample after short activation at 77 °C) (Figure 6b) caused no change in the chemisorbed occupancy, with a reduction of only 5% in the occupancy of the physisorbed site. Dynamic vacuum (10^–2^ mbar) at 25 °C for a further hour resulted in a reduction of 8% occupancy to the first physisorbed site and the second physisorbed site and no reduction in occupancy at the chemisorbed site. Increasing the temperature to 50 °C and applying a stronger dynamic vacuum (10^–6^ mbar) for a further 1 h resulted in a significant loss of occupancy at both physisorbed sites, with the occupancies dropping below 5%. The total occupancy of the chemisorbed site is also reduced by 25%. The excess electron density is too noisy to draw firm conclusions from (Figure S-3).

Taking the above results into account when evaluating laboratory procedures, purge treatments on MOF/composite materials probably only have a minimal effect on the chemisorbed NO and we can state that the NO-loaded MOF is broadly stable with respect to such conditions. However, it may have a larger effect on physisorbed NO, so care must be taken not to use too energetic conditions. This finding may explain why bulk samples activated between 80 and 177 °C and loaded with 1 bar of NO on a gravimetric rig, then exposed to 10^–1^ mbar vacuum for 30 min retain roughly 95% of the adsorbed NO (Figure 6c). However, the sample activated at 25 °C lost significantly more NO than any other sample after vacuum treatment (14%), suggesting that the NO may be bound more weakly to this sample. While the partially activated sample can still bind a high amount of NO through chemi- and physisorption, exposure to vacuum caused the removal of some of the physisorbed NO. However, this finding may be beneficial as the removal of physisorbed NO at low temperatures may allow for improved recyclability of the material, for instance, in NOx capture applications.

### Energies of Adsorption for NO in Partially Activated Ni-MOF-74

The energy of interaction between small molecule guests and MOFs is usually experimentally probed by variable temperature adsorption experiments. For fully activated MOFs, this is a relatively straightforward experiment. Unfortunately, for partially activated MOFs, this is an invalid experiment as varying the temperature changes the water content and the partition of water between the various sites, meaning that each adsorption temperature is done on a different material, which leads to incorrect results. To estimate the adsorption energies of NO on the MOFs, we therefore used density functional theory (DFT) calculations. These in themselves are complex calculations, as now both the nickel metal and the NO are open-shell species. Work by Smit and Neaton^46^ explains the complexities of calculating adsorption energies of small molecules on open metal sites in MOFs and how the errors compared to experimental work can be up to 30%. The energy of adsorption of NO on fully activated Ni-MOF-74 is measured at between 40 and 45 kJ/mol.^47^ Our calculations (see Supporting Information for details) result in values of 47 and 44 kJ/mol for the chemisorbed and physisorbed NO, respectively. The calculations likely overestimate the energies slightly (broadly consistent with the previous work of Smit) but do indicate that the physisorbed site, while being surprisingly strong, is weaker than the chemisorbed site, which is consistent with the structural work and the adsorption/desorption measurements described above.

## Discussion

By collecting in situ scXRD data of NO loading in Ni-MOF-74, we have uncovered a physisorbed binding site that exists only when water is also present in the material. This defies our previous expectations that assumed we could only achieve maximum NO loading if we had a fully activated MOF and had access to all the open metal sites. Instead, it seems that even when partially activating at mild temperatures of 80 °C, the remaining water allows for equivalent amounts of NO loading to be achieved by creating a secondary binding site through hydrogen bonding to metal-bound water molecules. This physisorbed site does appear to be more sensitive to vacuum and heat suggesting that the binding is weaker than binding to the metal site but is still strong enough to be beneficial, which is supported by calculations of the adsorption energies.

The calculations suggest that the physisorbed site has only a slightly lower binding energy than that of the chemisorbed site. However, the total amount of NO adsorbed is a balance between the energetics of the adsorption sites and the relative numbers of sites available for adsorption. As the activation temperature increases, the number of physisorption sites available decreases, but the number of chemisorption sites increases. At 80 °C, where the residual water content (Table 1) gives approximately 50% water-bound metal and 50% open metal sites, one should note that each chemisorbed water molecule produces more than one possible site for physisorption, whereas each open metal site can only bind one NO molecule. This means that after partial activation at 80 °C there are actually more physisorption sites available than chemisorption sites, leading to an increase in total NO adsorption compared with the fully activated sample. This explains why the NO adsorption capacity at room temperature and 1 bar of NO does not decrease upon lowering the activation temperature from 177 to 80 °C. Reducing the activation temperature further reduces the NO adsorption capacity because there is now remaining physisorbed water that competes for the physisorption sites, as can be seen in Table 1.

## Conclusions

This work shows the power of in situ scXRD experiments and the importance of thorough characterization to understand how MOFs bind gaseous guests in different conditions. Previous assumptions and models of gas binding in MOFs with open metal sites have focused on fully activated structures and binding to the open metal sites. It is only with detailed scXRD studies that we can observe there is in fact an additional binding site to be considered that accounts for the higher NO loading levels at lower activation temperatures. This knowledge allows new models and processes to be developed to maximize gas binding within MOFs and MOF composites.

## Experimental Section

### Synthesis

Single crystals of Ni-MOF-74 were synthesized using the procedure published by Vornholt et al.^32^ Nickel acetate tetrahydrate (1 mmol) was dissolved in water (30 mL) and added to a Teflon liner (50 mL). 2,5-Dihydroxyterephthalic acid (0.5 mmol) and 4,6-dihydroxyterephthalic acid (0.5 mmol) were added to the liner and left to stir for 15 min. The liner was capped, sealed in an autoclave, and placed in the oven for 3 days at 130 °C. Yellow-brown, rectangular rods of Ni-MOF-74 were obtained after filtration.

### Single-Crystal Experiments

In situ gas cell diffraction experiments on single crystals were carried out on a four-circle Newport diffractometer equipped with a Dectris Pilatus 300 K detector in the I19-2 beamline, Diamond Light Source. A wavelength of 0.48590 Å (Ag K-edge) was utilized to give a complete data set from a single 340° phi sweep (1700 images, 0.2°/image). Selected crystals were mounted with a mitogen mount (50 μm) and were secured with a nondiffracting two component epoxy glue (LOCKTITE DOUBLE BUBBLE). Care was taken to use as little glue as possible to avoid blocking any channels to ensure good gas transport through the crystal. For gas cell experiments, the crystal mount was inserted into a preassembled gas cell, with super glue used to hold the mount securely in place in the gas cell capillary. The gas cell was then sealed using the Swagelok mechanism and leak tested.

The fully activated sample was obtained by exposing a selected crystal to 177 °C in vacuo (3.1 × 10^–6^ mbar at the pump) for 4 h. The crystal was then cooled to 25 °C in vacuo. The gas cell was then filled with a 2 bar NO atmosphere and the sample was left to equilibrate for 30 min.

To collect the gas adsorption isotherm, a crystal was first activated at 80 °C in vacuo (3.1 × 10^–6^ mbar at the pump) for 9 h. The crystal temperature was then reduced to 25 °C. The NO pressure was increased sequentially from vacuum to 0.01, 0.05, 0.1, 0.2, 0.4, 0.8, 1.6, 2.0 bar, with a scan taken after 5 and 30 min at each pressure value. The cell was flushed with a vacuum/argon cycle three times before being left under vacuum for 3 h at 25 °C, then 1 h at 50 °C.

A second gas adsorption isotherm was generated by activating a crystal at 77 °C in vacuo (3.1 × 10^–6^ mbar at the pump) for 4 h. NO was introduced at a pressure of 0.08 bar. The crystal temperature was then reduced to 25 °C and the NO pressure was sequentially increased to 0.2, 0.39, 1.0, and 2.0 bar, with a scan taken at each pressure after 30 min. The cell was flushed with a vacuum/argon cycle three times, placed under vacuum (10^–2^ mbar) at 25 °C for 1 h, then under a higher vacuum (10^–6^) at 50 °C for 1 h.

Data collection were setup using the general data acquisition (GDA) software and were automatically processed using Xia2 ^48^ with DIALS^49^ routines. All samples were indexed with DIALS 3.6.2-g16e93f55b-release.^49^ Visualization was performed using Olex2 1.5 GUI^44^ and ShelXT 2014/4 ^50^ for solving and ShelXL 2018/3 ^51^ for refinement. Obtained crystal structures were visualized using the CrystalMaker software kit.^52^ Special refinement details can be found in the Supporting Information.

### Adsorption Experiments

N_2_ adsorption isotherms were recorded on a Micromeritics Tristar II Surface Area and Porosity Instrument. Samples were added to a frit tube and activated in vacuo (150, 80, 50, or 25 °C, ∼3 × 10^–4^ mbar, 16 h) prior to the measurement.

The nitric oxide adsorption–desorption measurements were collected using a bespoke gravimetric adsorption system. This system consists of a highly sensitive microbalance (sensitivity of 0.1 μg and reproducibility of 0.01%) and a pressure gauge. Each sample was activated at 177, 150, 80, 50, or 25 °C under a pressure of ×10^–4^ mbar overnight until no further mass loss was observed and the mass loss recorded. The samples were cooled to 25 °C by using a water bath (temperature accuracy of 0.02 °C). Nitric oxide gas was incrementally introduced to the system to produce isotherms or directly to 1 bar, and after each introduction, the recorded mass of the sample was allowed to stabilize before the next NO addition was made. The adsorption process was deemed to be complete when the introduced pressure of NO was equal to the atmospheric pressure. The desorption is measured by exposing the sample to reduction in pressure to ×10^–1^ mbar.

## Supplementary Material



## Data Availability

The research data supporting this publication can be accessed at 10.17630/c6969b9e-62dc-4611-9336-ae49c58cc25d.

## Notes

Refinement details and asymmetric units for each structure presented; N_2_ adsorption isotherms for Ni-MOF-74 after different activation conditions; NO isotherm for loading in Ni-MOF-74 after short activation (PDF)

## Abbreviations

MOF: metal–organic framework
NO: nitric oxide
PXRD: powder X-ray diffraction
scXRD: single-crystal X-ray diffraction
dhtp: dihydroxyterephthalic acid
