# Supplementary material for: In Situ Single-Crystal X‑ray Diffraction Studies of an Anomalous Nitric Oxide Adsorption in a Partially Activated Metal–Organic Framework
Source: J Am Chem Soc. 2025 Aug 14;147(34):31260–9. doi: 10.1021/jacs.5c10395 (PMC12395409; doi:10.1021/jacs.5c10395)
Supplement: Supplementary file 1 [file ja5c10395_si_001.pdf]

In situ single-crystal X-ray diffraction studies of anomalous nitric oxide adsorption in a partially activated metal-organic framework

Supplementary Information

Russell M. Main, [1*] Romy Ettlinger, [1,2] Tia K. Tajnšek[1,3], Deborah A. Brako-Amoafo[1,4], Maximillian G. Stanzione[1], Morven J. Duncan[1], Philip Ettlinger[1], Gaynor B. Lawrence[1], Mark R. Warren[5] Christopher J. Heard[4] and Russell E. Morris[1*]

[1] EaStCHEM School of Chemistry, Purdie Building, North Haugh, St Andrews KY16 9ST, UK.

[2] TUM School of Natural Sciences, Technical University of Munich, Lichtenbergstr.4; 85748 Garching b. München, Germany.

[3] National Institute of Chemistry, Hajdrihova 19, 1000, Ljubljana, Slovenia

[4] Department of Physical and Macromolecular Chemistry, Charles Univsersity, Hlavova 8, 12800 Prague 2, Czech Republic.

[5] Diamond Light Source Ltd, Diamond House, Harwell Science & Innovation Campus, Didcot, OX11 0DE, UK.

Contents

Supplementary isotherms 2

Supplementary crystallographic information 5

Supplementary refinement details 5

Asymmetric units 7

Computer calculations 9

Supplementary references 10

Supplementary isotherms

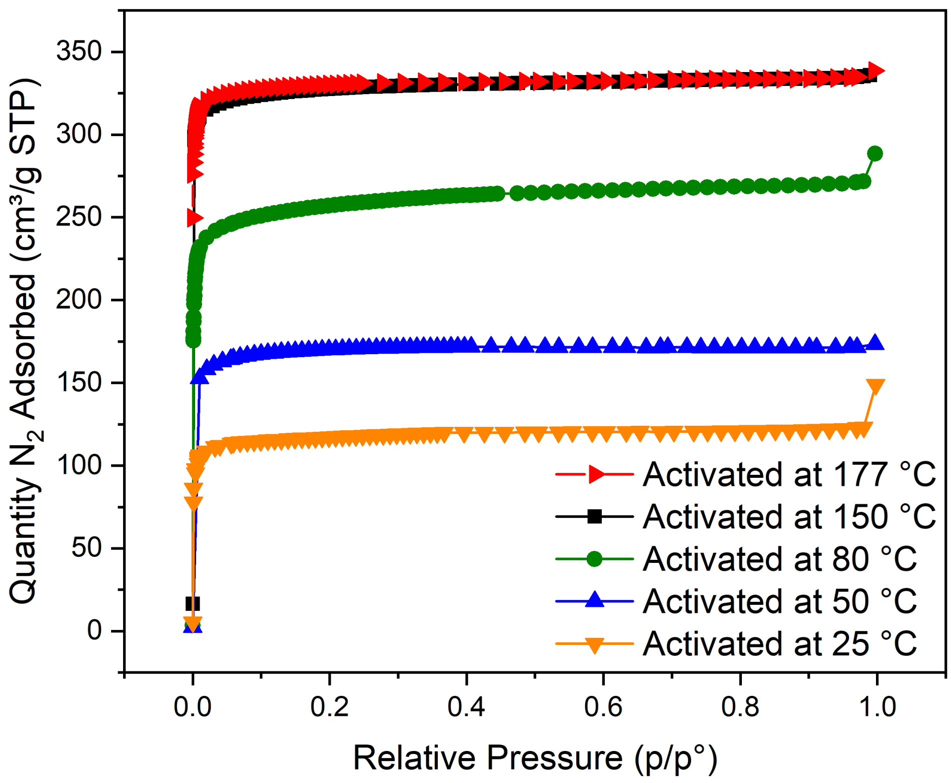

Figure S-1: a) N_2 adsorption isotherms at 77 K and for Ni-MOF-74 after overnight activation, under vacuum at 177 °C (red), 150 °C (black), 80 °C (green), 50 °C (blue) and 25 °C (orange).

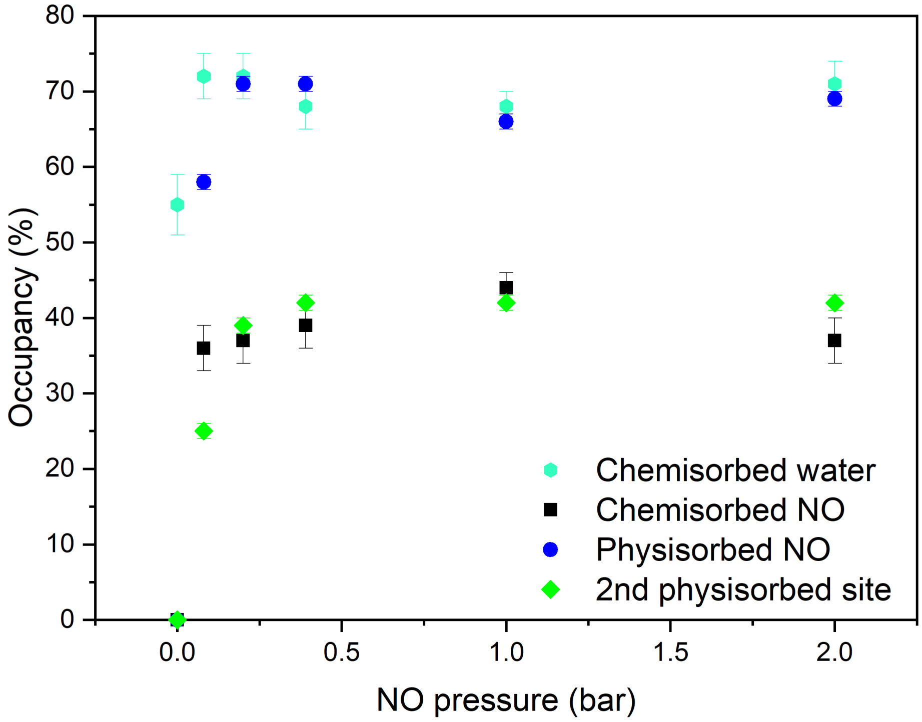

Figure S-2: Isotherm of occupancy vs NO pressure of NO bound at the metal site (black), physisorbed NO (blue) and the occupancy of an oxygen in a second binding site (green) for Ni-MOF-74 after short activation at 77 °C for 3 h.

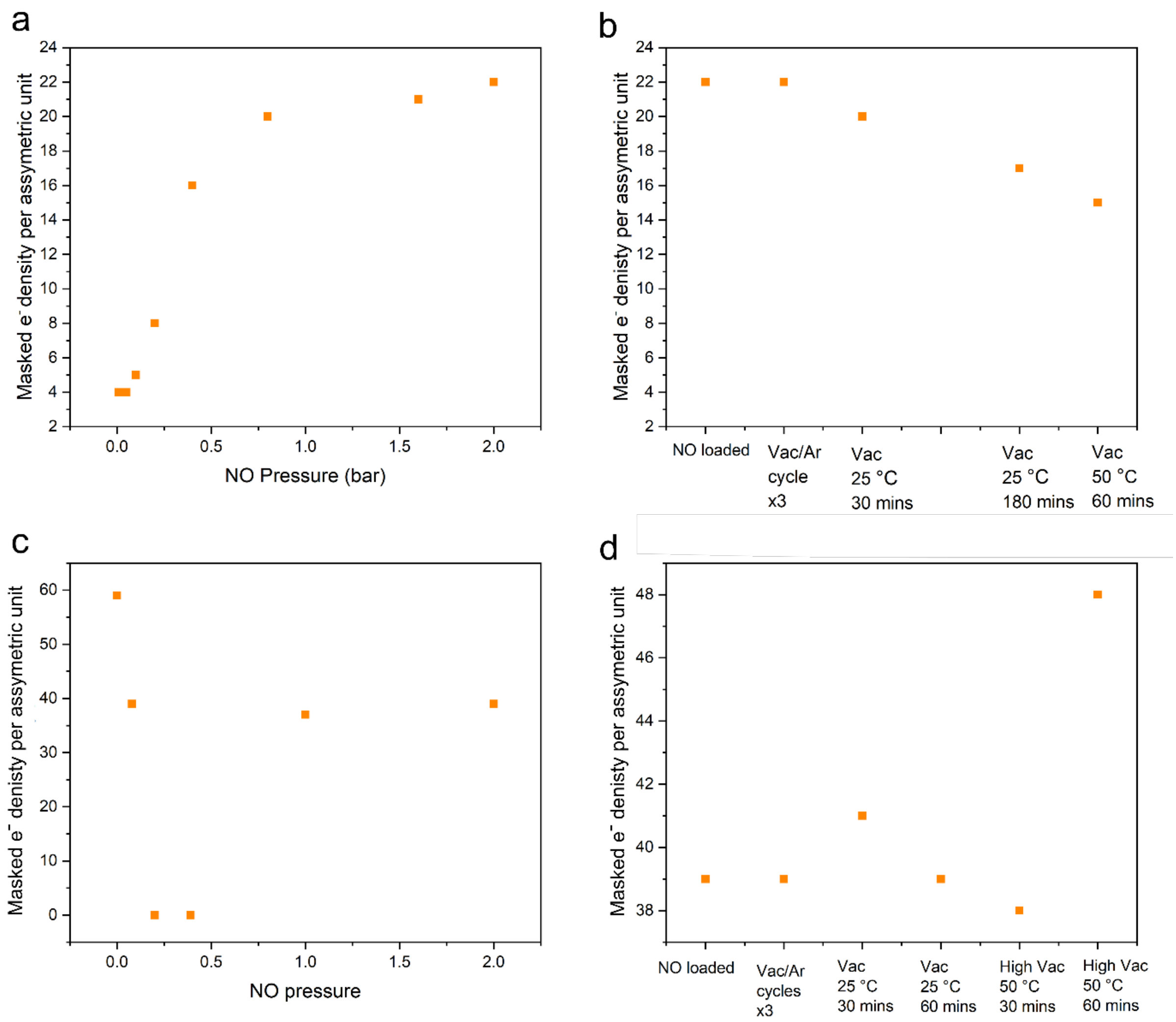

Figure S-3: The electron density as calculated with Olex2 mask command with a 1.2 Å probe. A) NO loading isotherm of Ni-MOF-74 after activation at 80 °C for 9 h. b) The sample in a) subject to a series of vacuum conditions. C) NO loading isotherm of Ni-MOF-74 after activation at 77 °C for 3 h. d) The sample in c) subject to a series of vacuum conditions.

Supplementary crystallographic information

Supplementary refinement details

To generate the first NO loading isotherm, a similar model was used for each sample to improve comparability between the data points. The Ni-MOF-74 showed the expected $R\bar{3}$ symmetry, however the crystal was twinned which was accounted for in the refinement with a $\begin{matrix} 0 & -1 & 0 \\ -1 & 0 & 0 \\ 0 & 0 & -1 \end{matrix}$ twin law which considerably improved the final agreement factors. The metal bound NO was modelled with Exyz position and Uiso constraints, with only the occupancies freely refinable. To account for the high initial water occupancy an oxygen atom with 63% occupancy was also modelled at the metal site, with the same Uiso as the N. Only one oxygen site in the chemisorbed NO with a constrained Uiso of 0.06 and subject to DANG and DFIX restraints. The final refinement models less of the disorder of the NO than in previous work,[1,2] but including such complex models, especially with the addition of metal-bound water, did not improve the crystallography and so were not used. The physisorbed NO also had constrained Uiso of 0.05 for both atoms and the position was constrained at lower pressures, based on the position refined at 1 and 2 bar. This N-O bond length was restrained with a DFIX restraint of 1.3 Å, this is longer than the gas bond length[3] but is similar to that seen in other single crydtal refinements. For the samples under vacuum conditions the metal bound atom was modelled as an oxygen with free occupancy. For the samples were this occupancy >100% it was fixed to 100% in the final CIFs. For this crystal under vacuum conditions the metal bound atom was modelled as nitrogen with free occupancy. This occupancy was allowed to be >100% to include the additional electron density from the oxygen of the chemisorbed water.

A similar model was used for the second NO loading isotherm. The metal bound occupancies of O and N were constrained with a EXYZ command and their occupancies restrained with SUMP commands. At least two oxygen sites could be modelled at the metal bound NO with more sites refinable at higher pressures, these had a constrained Uiso of 0.06 and subject to DANG and DFIX restraints. The physisorbed N-O bond length was restrained with a DFIX restraint of 1.3 Å. A second physisorbed site could be modelled with an oxygen atom with constrained Uiso of 0.05. For this crystal under vacuum conditions the metal bound atom was modelled as an oxygen with free occupancy. For the samples where this occupancy >100% it was fixed to 100% in the final CIFs.

R_1 values and CCDC numbers for each structure presented can be found in Table S-1-S-5.

Table S-1:Crystallographic information and CCDC number for the Ni-MOF-74 crystal activated at 177 °C.

Sample conditions	Ow / %	Chemisorbed NO / %	Masked e^- density / ASU	R1	CCDC number
Activated at 450 K and cooled to 300 K	17.4(10)	0	0	8.29	2411474
2 bar NO	17	80.4(8)	0	4.16	2411475

Table S-2: Crystallographic information and CCDC number for the Ni-MOF-74 crystal activated at 80 °C for 9h.

Sample conditions	Ow / %	Chemisorbed NO / Nc%	Physisorbed NO / Op%	Masked e^- density / ASU	R1	CCDC number
Activated 353 K, 9h	53.3(5)	0	0	4	4.29	2411476
Cooled to 300 K	59.2(5)	0	0	4	4.35	2303169
0.01 bar NO	63	18.0(6)	7.8(5)	3	4.54	2303170
0.005 bar NO	63	28.7(6)	11.6(5)	4	4.52	2303171
0.1 bar NO	63	35.8(6)	22.7(6)	5	4.61	2303172
0.2 bar NO	63	44.2(7)	34.7(7)	8	5.15	2303173
0.4 bar NO	63	44.3(7)	42.2(7)	16	5.51	2303174
0.8 bar NO	63	44.7(8)	44.6(7)	20	5.78	2303175
1.6 bar NO	63	42.7(8)	45.5(7)	21	5.76	2303176
2 bar NO	63	42.5(8)	43.1(7)	22	5.87	2303177

Table S-3:Crystallographic information and CCDC number for the crystal in Table S-2 subject to vacuum conditions.

Sample conditions	N chemisorbed / %	O physisorbed / %	Masked e^- density / ASU	R1	CCDC number
3x Vacuum/Ar purge	102.5(11)	43.6(10)	22	7.58	2303180
30 mins vac, 300 K	101.8(11)	39.7(9)	20	7.61	2303181
180 mins vac, 300 K	95.1(11)	27.5(9)	17	7.29	2303182
60 mins vac 323 K	81.8(9)	13.6(7)	15	6.66	2303183

Table S-4: Crystallographic information and CCDC number for the Ni-MOF-74 crystal activated at 77 °C for 3h.

Sample conditions	Ow / %	NO chemisorbed / Nc%	NO physisorbed / Op%	2nd physisorbed site / O%	Masked e^- density / ASU	R1	CCDC number
Activated 350 K	54.5(4)	0	0	0	59	4.46	2384936
0.08 bar NO 350 K	54.7(10)	24.8(13)	0	0	51	4.65	2384937
0.08 bar NO 300 K	72(3)	36(3)	57.8(6)	24.5(6)	39	4.90	2384938

0.2 bar NO	72(3)	37(3)	69.1(7)	38.6(6)	0	5.57	2384939
0.39 bar NO	68(3)	39(3)	71.3(6)	41.6(6)	0	5.40	2384940
1 bar NO	78(2)	33(3)	71.5(6)	41.7(5)	37	4.88	2384941
2 bar NO	71(3)	38(3)	65.6(7)	41.9(6)	39	5.48	2384942

Table S-5:Crystallographic information and CCDC number for the crystal in Table S-4 subject to vacuum conditions.

Sample conditions	Chemisorbed / O%	Physisorbed / O%	2nd physisorbed site / O%	Masked e^- density / ASU	R1	CCDC number
3x Vacuum/Ar purge	106.4(6)	64.1(6)	38.8(6)	39	5.24	2384931
Low Vac 25 °C 30 m	104.8(6)	58.5(6)	32.1(6)	41	5.23	2384932
Low Vac 25 °C 60 m	105.5(6)	56.8(7)	30.9(6)	39	5.41	2384933
High Vac 50 °C 30 m	104.3(6)	57.0(6)	31.0(6)	39	5.39	2384934
High Vac 50 °C 60 m	83.0(6)	4.1(5)	0	48	4.91	2384935

Asymmetric units

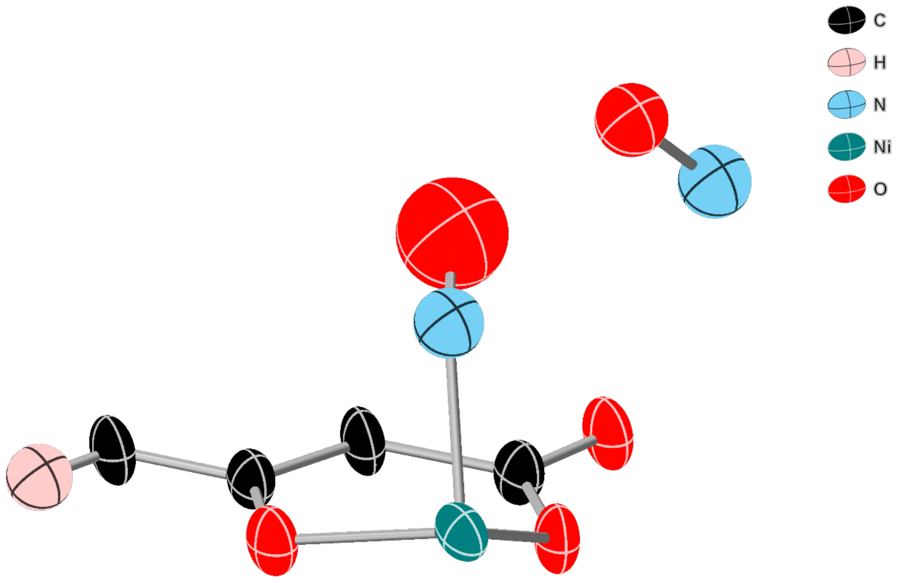

Figure S-4: Asymmetric unit with 50% probability ellipsoids of the model used for NO loaded Ni-MOF-74 after activation at 80 °C for 9 h.

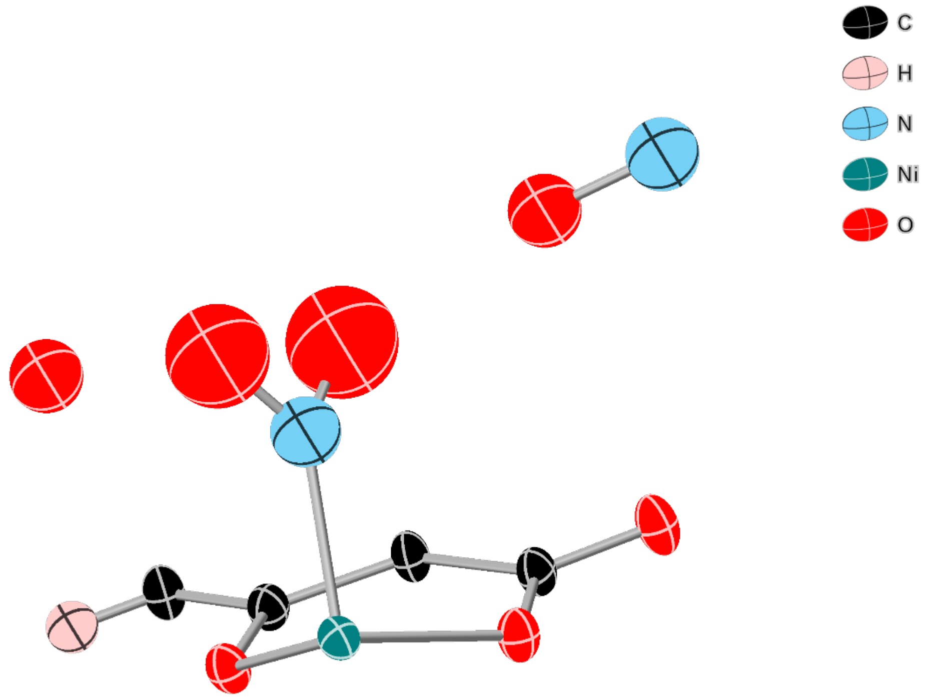

Figure S-5: Asymmetric unit with 50% probability ellipsoids of the model used for NO loaded Ni-MOF-74 after activation at 77 °C for 3 h.

Computer calculations

Spin-polarized density functional calculations were performed to identify the adsorption and co-adsorption propensity of nitric oxide, into both fully and partially activated Ni-MOF-74. We considered models in which 18 NO molecules per unit cell are adsorbed either to the bare Ni-MOF-74 or the water loaded (18 H_2O molecules per unit cell) Ni-MOF-74.

Adsorption energies (E_{ads}) were calculated using equation [1],

$$E_{ads} = \frac{E_{X@MOF74} - E_{MOF74} - nE_X(g)}{n} \qquad [1]$$

where X is the adsorbing species (NO) and n is the number of molecules sustained by the MOF. E_{ads} of NO was determined to be -58 kJ mol^{-1} per molecule. The co-adsorption energy ($E_{co\text{-}ads}$) of NO into the water pre-loaded MOF was determined to be moderately lower (-51 kJ mol^{-1}), according to equation [2],

$$E_{co-ads} = \frac{E_{(NO)n@H2O-MOF74} - E_{H2O-MOF74} - nE_{NO}(g)}{n} \qquad [2]$$

While the full consideration of all possible NO locations and H_2O/NO ratios are beyond the scope of the current work, these findings are sufficient to suggest that there are additional stable binding possibilities available for NO into the partially activated MOF, in agreement with the conclusions from the scXRD measurements.

Experimental

Spin-polarization calculations were performed within the Vienna Ab Initio Package (version 6.3.2), at the generalised gradient level of density functional theory, utilizing the Perdew-Burke-Ernzerhof exchange correlation functional, augmented with the Grimme D3 dispersion correction with Becke-Johnson damping, to capture the dispersive contribution to molecular binding. A Hubbard +U correction was added to the d orbitals of the Nickel atoms, with an effective U parameter of 6.4 eV, using the Dudarev approach, to model 3d electron correlation effects, in accordance with prior calculations[4] aimed at reproducing experimental formation energies of Ni_2O_3 from the nickel monoxide.[5] The magnetic moments of nickel atoms were initialised according to a chain-by-chain antiferromagnetic ordering, in line with previous calculations on related systems.[6,7]

Local geometry optimization of atoms was undertaken using the conjugate gradient algorithm, with a force convergence criterion of 1x10^{-3} eV / Å. Wavefunctions were described with a plane wave basis, and expanded to a kinetic energy cutoff of 850 eV. Electronic convergence within self consistent loops was achieved with a threshold of 1x10^{-8} eV., with Gaussian smearing of the Fermi-Dirac distribution, with a smearing width of 0.1 eV, to aid convergence. Electronic sampling of the Brillouin zone was undertaken with a kpoint grid of 1x1x2.

Supplementary references

(1) Main, R. M.; Vornholt, S. M.; Rice, C. M.; Elliott, C.; Russell, S. E.; Kerr, P. J.; Warren, M. R.; Morris, R. E. In Situ Single-Crystal Synchrotron X-Ray Diffraction Studies of Biologically Active Gases in Metal-Organic Frameworks. *Commun. Chem.* **2023**, *6* (1), 1–7. https://doi.org/10.1038/s42004-023-00845-1.

(2) Vornholt, S. M.; Elliott, C. G.; Rice, C. M.; Russell, S. E.; Kerr, P. J.; Rainer, D. N.; Mazur, M.; Warren, M. R.; Wheatley, P. S.; Morris, R. E. Controlled Synthesis of Large Single Crystals of Metal-Organic Framework CPO-27-Ni Prepared by a Modulation Approach: In Situ Single-Crystal X-Ray Diffraction Studies. *Chem. - A Eur. J.* **2021**, *27* (33), 8537–8546. https://doi.org/10.1002/chem.202100528.

(3) *Diatomic Spectral Database | NIST*. https://www.nist.gov/pml/diatomic-spectral-database (accessed 2022-04-13).

(4) Lee, K.; Howe, J. D.; Lin, L. C.; Smit, B.; Neaton, J. B. Small-Molecule Adsorption in Open-Site Metal-Organic Frameworks: A Systematic Density Functional Theory Study for Rational Design. *Chem. Mater.* **2015**, *27* (3), 668-678. DOI: 10.1021/cm502760q.

(5) Wang, L.; Maxisch, T.; Ceder, G. Oxidation energies of transition metal oxides within the GGA+U framework. *Phys Rev B* **2006**, *73* (19). DOI: ARTN 195107 10.1103/PhysRevB.73.195107.

(6) Mukoyoshi, M.; Maesato, M.; Kawaguchi, S.; Kubota, Y.; Cho, K.; Kitagawa, Y.; Kitagawa, H. Systematic Tuning of the Magnetic Properties in Mixed-Metal MOF-74. *Inorg Chem* **2022**, *61* (19), 7226-7230. DOI: 10.1021/acs.inorgchem.2c00646.

(7) Dietzel, P. D. C.; Morita, Y.; Blom, R.; Fjellvåg, H. An in situ high-temperature single-crystal investigation of a dehydrated metal-organic framework compound and field-induced magnetization of one-dimensional metaloxygen chains. *Angew Chem Int Edit* **2005**, *44* (39), 6354-6358. DOI: 10.1002/anie.200501508.
